# Safety, efficacy, and survival of different transarterial chemoembolization techniques in the management of unresectable hepatocellular carcinoma: a comparative single-center analysis

**DOI:** 10.1007/s00432-024-05722-5

**Published:** 2024-05-06

**Authors:** Philipp Schindler, Daniel Kaldewey, Florian Rennebaum, Jonel Trebicka, Andreas Pascher, Moritz Wildgruber, Michael Köhler, Max Masthoff

**Affiliations:** 1 Clinic for Radiology, University of Münster, Albert-Schweitzer-Campus 1, 48149 Münster, Germany; 2 Department of Internal Medicine B, University of Münster, Münster, Germany; 3 Department of General, Visceral and Transplant Surgery, University of Münster, Münster, Germany; 4 Department of Radiology, LMU Munich, Munich, Germany

**Keywords:** TACE, DEB-TACE, DSM-TACE, cTACE, HCC

## Abstract

**Purpose:**

Transarterial chemoembolization (TACE) has become the standard of care for the treatment of intermediate-stage hepatocellular carcinoma (HCC). However, current clinical practice guidelines lack consensus on the best selection of a specific TACE technique. This study aims to compare safety, tumor response, and progression-free survival (PFS) of conventional TACE (cTACE), drug-eluting bead TACE (DEB-TACE), and degradable starch microsphere TACE (DSM-TACE).

**Methods:**

This retrospective study included *n *= 192 patients with HCC who underwent first TACE with unbiased follow-up at 4–6 weeks at our center between 2008 and 2021. Eligibility for TACE was BCLC intermediate stage B, bridging/down-staging (B/D) to liver transplantation (LT), or any other stage when patients were not suitable for resection, LT, local ablation, or systemic therapy. Patients were grouped into three cohorts (*n *= 45 cTACE, *n *= 84 DEB-TACE, *n *= 63 DSM-TACE), and further categorized by TACE indication (B/D or palliative). Liver function and adverse events, response assessed by the modified response evaluation criteria in solid tumors (mRECIST) 4–6 weeks post-TACE and PFS were analyzed.

**Results:**

There were no significant differences in age, gender distribution, BCLC stage, or etiology of liver disease among the three TACE groups, even in the B/D or palliative subgroups. DEB-TACE induced slight increases in bilirubin in the palliative subgroup and in lactate dehydrogenase in the entire cohort 4–6 weeks post-TACE, and more adverse events in the palliative subgroup. DEB-TACE and DSM-TACE showed significantly higher disease control rates (complete and partial response, stable disease) compared to cTACE, especially in the B/D setting (*p* < 0.05). There was no significant difference in PFS between the groups [median PFS (months): cTACE, 10.0 vs. DEB, 7.0 vs. DSM, 10.0; *p* = 0.436].

**Conclusion:**

Our study provides valuable perspectives in the decision-making for a specific TACE technique: DEB-TACE and DSM-TACE showed improved tumor response. DEB-TACE showed a prolonged impact on liver function and more side effects, so patients with impaired liver function should be more strictly selected, especially in the palliative subgroup.

## Introduction

Transarterial chemoembolization (TACE) has emerged as one of the standard treatments for hepatocellular carcinoma (HCC), offering both palliative and curative benefits (Müller et al. 2021). This minimally invasive locoregional endovascular therapy combines the dual effects of delivering chemotherapeutic agents directly to the tumor site while inducing ischemic necrosis through embolic agents, making it a cornerstone in the treatment algorithm for HCC (Reig et al. 2022).

The indication for TACE typically includes patients with intermediate-stage HCC, as defined by the Barcelona clinic liver cancer (BCLC) staging system, for whom curative options such as surgical resection or local ablation are not an option and systemic therapies are contraindicated or have resulted in tumor progression (Reig et al. 2022). In addition to its use in the palliative setting for intermediate tumor stages, TACE is used as a bridging treatment to liver transplantation (LT) to control local tumor growth or for down-staging the patient’s tumor burden aiming to reach eligibility for LT (Claasen et al. 2023).

Despite its established role, the landscape of TACE procedures has evolved considerably over the years, resulting in a variety of techniques and agents (Saghafian Larijani et al. 2022). Besides conventional TACE (cTACE), drug-eluting bead TACE (DEB-TACE) and degradable starch microsphere TACE (DSM-TACE) have received considerable attention for their unique characteristics (Saghafian Larijani et al. 2022).

cTACE is a traditional approach in which a chemotherapy agent mixed with an embolic agent is injected directly into the HCC feeding artery. The embolic agent blocks blood flow to the tumor, while the chemotherapy remains trapped inside the tumor. While cTACE has a long history of use and been proven effective in treating unresectable HCC, it has limitations related to the precision of drug delivery and variable response rates (Llovet and Bruix 2003). DEB-TACE uses drug-eluting microspheres loaded with chemotherapeutic agents. These microspheres are selectively delivered to the vessels feeding the tumor. Controlled drug release from the beads increases drug concentrations within the tumor while minimizing systemic exposure (Zarisfi et al. 2022). DEB-TACE has gained popularity due to its potential for improved drug delivery and reduced side effects (Lammer et al. 2010). DSM-TACE uses degradable starch microspheres in combination with chemotherapy as an embolic agent. These microspheres dissolve gradually, providing sustained drug release within the tumor. DSM-TACE offers the advantage of prolonged drug exposure while minimizing the risk of embolization-related complications (Iezzi et al. 2019).

However, current clinical practice guidelines do not provide an algorithm for the optimal choice of the TACE technique to be used, and few studies have directly compared these techniques within a single cohort (Marrero et al. 2018; Angeli et al. 2018; Lucatelli et al. 2021; Mohr et al. 2022). Therefore, there is a compelling need for comprehensive analyses evaluating safety, efficacy, and survival outcomes associated with these different TACE techniques. We evaluated the first TACE efficacy in terms of tumor response, impact on liver function, incidence of adverse events, and progression-free survival (PFS).

## Methods

### Study design

The study was conducted as a retrospective, single-center, observational study at a tertiary care academic medical center in Germany. The study was conducted in accordance with the tenets of the Declaration of Helsinki (as revised in 2013). The study was approved by the local ethics committee (ID: 2020-495-f-S). Informed consent was not obtained from patients due to the retrospective nature of this study.

### Patient selection

All patients with HCC who underwent at least one TACE (*n *= 277), identified from all patients with TACE (*n *= 321) in our center between 2008 and 2021, were reviewed. All patients undergoing TACE were approved by the interdisciplinary gastrointestinal tumor board. Diagnosis was based on European Association for the Study of the Liver (EASL) clinical practice guidelines (Angeli et al. 2018). Patients were assigned to TACE at BCLC intermediate stage B, for bridging/down-staging (B/D) to liver transplantation (LT), or at any other stage when patients were ineligible for resection, LT, local ablation, or systemic therapy.

**Fig. 1:**
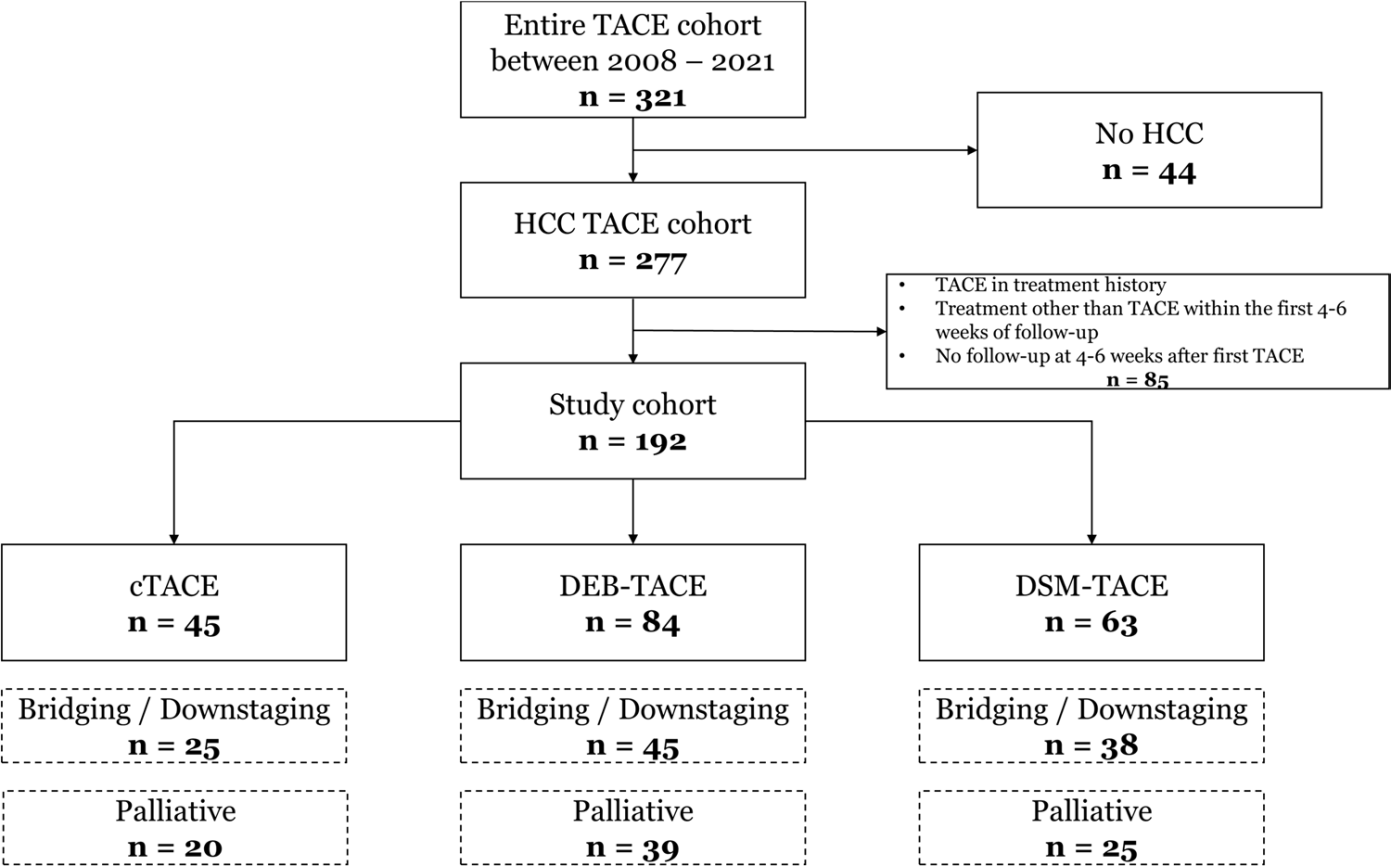
Flow chart of study design

We defined the following inclusion criteria: no TACE in treatment history and absence of treatment other than TACE within the first 4–6 weeks of follow-up to avoid selection bias, and availability of at least one follow-up after first TACE at 4–6 weeks. A total of 192 patients were thus finally enrolled in this study. Patients were divided into three cohorts based on the first TACE procedure used: *n *= 45 received cTACE, *n *= 84 received DEB-TACE, and *n *= 63 received DSM-TACE, with the decision on the procedure made by the interventional radiologist with at least 5 years of experience. Subgroup analysis was performed regarding the indication for TACE: B/D, non-B/D = palliative including patients ineligible for resection, LT, local ablation, or systemic therapy. Detailed study design is shown in Fig. 1.

### TACE procedures

The goal of TACE in HCC is to locally treat the tumor tissue by transarterial administration of a combination of anticancer/chemotherapeutic drugs and embolic agents to hinder washout of the chemotherapeutic agents and reduce tumor blood supply, supporting intratumoral necrosis. Although introduced as early as 1979 with plenty subsequent studies contributing to the standard-of-care establishment in nearly all guidelines (Marrero et al. 2018; Angeli et al. 2018; Lucatelli et al. 2021), there is a variety of TACE procedures available and performed as global surveys revealed (Young et al. 2019; Craig et al. 2019). In this study, every TACE procedure was performed by a board-certified and experienced (> 4 years) interventional radiologists trained in all three TACE procedures. CT with at least arterial and venous phase or MRI with dynamic contrast-enhanced sequences not older than 4 weeks was used for procedure planning. Hepatic tumor burden (I: 0–25%, II: 26–50%, III: > 50%) was defined as total volume of tumor tissue over total liver volume derived from three-dimensional imaging (CT or MRI) prior to TACE. cTACE, DEB-TACE, and DSM-TACE were performed according to CIRSE standard-of practice guidelines (Lucatelli et al. 2021). Briefly, after 5F retrograde trans-femoral access contrast pump-based angiograms from mesenteric artery, celiac trunk and/or abdominal aorta (flow rate 4 ml/s, volume 20 ml or 15 ml/s with 20 ml volume, respectively) were performed to identify tumor arterial supply. Subsequently, a coaxial micro-catheter system (2.0–2.4F) was placed as supra-selective to the tumor as possible. Selectivity in this study was defined as (1) super-selective: micro-catheter tip placed with only tumor vessels contrasted in control angiogram, (2) selective: micro-catheter tip placed with tumor vessels and only one non-tumor side branch (sub-segmental) contrasted in control angiogram, or (3) unselective: micro-catheter tip placed with tumor vessels and more than one non-tumor side branch (segmental/lobar) contrasted in control angiogram. If necessary, cone-beam CT (CBCT) was performed to control micro-catheter tip position or detect additional/variant tumor supply feeders. After micro-catheter positioning, drug delivery was performed with a 1–3 ml syringe in a flow-control fashion according to the following: For cTACE, a mixture of 50 mg of Doxorubicin was slowly mixed with lipiodol (Guerbet, France) in a water-in-oil (W/O) emulsion (1:2, 10 ml of lipiodol). Meanwhile, for DEB-TACE, 100 µm embozene microspheres (Varian Medical Systems, USA) were loaded with 50 mg of Doxorubicin according to manufacturer instructions. For DSM-TACE, 50 µm degradable starch microspheres (Embocept, Pharmacept, Germany) were mixed with 50 mg of Doxorubicin. TACE procedures end point was blood stasis in tumor feeding vessels. Accumulation of embolization material within the HCC lesions was finally confirmed with CBCT. Peri-procedural medication included analgesia (7.5 mg piritramide i.v.) and anti-emetics (4 mg ondansetron i.v.).

### Data collection and follow-up

All patient and procedural data, including laboratory parameters and follow-up/survival data, were collected retrospectively from the electronic medical record and the picture archiving and communications system (PACS). Patients were evaluated at baseline and 4 to 6 weeks post-TACE.

#### Response assessment

Patients underwent response assessment at 4 to 6 weeks after the first TACE without intermediate treatment according to the modified response evaluation criteria in solid tumors (mRECIST) using diagnostic CT scans or MRI if CT was contraindicated (Lencioni and Llovet 2010). In addition, we defined so-called "TACE lesions” as a HCC lesion, which was treated by TACE. “TACE lesions” may differ from “target lesions” defined by the standards of mRECIST, exemplarily if only one (out of two definable mRECIST “target lesions”) was addressed by the first TACE procedure due to dose limits. Response assessment was sub-divided into response (complete and partial response) and disease control rate (complete and partial response, and stable disease). Other assessments were performed according to current guidelines and the decision of the tumor board based on response to therapy and possible further TACE, radio-embolization (TARE), or systemic therapies.

#### Laboratory and adverse events

Liver function changes were monitored, where changes are expressed as delta (Δ), with positive values indicating an increase and negative values indicating a decrease 48 to 72 h and 4 to 6 weeks after TACE.

Adverse events (AE) were recorded according to the Cardiovascular and Interventional Radiological Society of Europe (CIRSE) Quality Assurance Document and Standards for Classification of Complications (The CIRSE Classification System) (Filippiadis et al. 2017). Here, CIRSE grade 1–3 summarize AE that required no additional therapy (grade 1), AE that required prolonged hospital stay > 48 h beyond the normal course (grade 2), and AE with additional post-procedure therapy or additional prolonged hospital stay > 48 h (grade 3). Meanwhile, CIRSE grade 4–6 summarize serious AE (SAE) with resulting mild (grade 4) or severe sequelae (grade 5) up to death (grade 6). Duration of the hospital stay and rate of patients with prolonged hospital stay > 48 h beyond the normal course (CIRSE AE grade 3) were monitored. Post-embolization syndrome events were monitored, including clinical symptoms, such as fever, nausea, and abdominal pain, beyond the normal post-procedure course requiring for additional therapy(De Baere et al. 2016; Lu et al. 2021).

#### Survival

PFS was calculated from the date of the first TACE procedure to radiologic progression, LT, or death due to any cause, whichever occurred first. For patients who have not progressed, transplanted, or died by the clinical cut-off date for analysis (2021-DEC-31), PFS will be censored at the date of the last adequate disease assessment. Further TACE cycles, including other techniques, as well as other locoregional therapies such as radioembolization (TARE) or systemic therapies were not a reason for censoring.

#### Bridging/downstaging and liver transplantation

For the B/D subgroup, data on listing after the first TACE and, in the case of listing, data on liver transplantation with time from the first TACE to liver transplantation were collected.

### Statistical analysis

Data are presented as total number and percentage, mean and standard deviation, median and range, or 95% confidence interval (CI), as appropriate. Statistical analysis was performed using Chi-Square test with Bonferroni post hoc test for categorical and ANOVA with Sidak post hoc test for continuous variables. Kaplan–Meier survival analysis was used to estimate PFS, and the log-rank test was used to compare survival curves among the three TACE procedures. P values < 0.05 were considered statistically significant. Statistical analysis was performed using SPSS Statistics version 29 (SPSS Inc., Chicago, IL, USA).

## Results

### Patient demographics

The demographic characteristics of the study population are summarized in Table 1. There were no significant differences in age, gender distribution, BCLC stage, or etiology of liver disease among the three TACE groups, even in the B/D or palliative subgroups. 108 patients (56.2%) were assigned to the B/D subgroup, of which *n *= 62 (57.4%) were within the Milan criteria. They had an overall lower mean sum of target lesion diameter compared to the palliative subgroup although with no difference in TACE technique. There were also no significant differences between groups with respect to catheter position selectivity, which indicates homogeneity of the groups and good comparability.

**Table 1:** Baseline patient characteristics of the entire study cohort and subgroups related to the indication für TACE

	Entire cohort					Bridging/downstaging					Palliative				
	Total	cTACE	DEB	DSM	*p*	Total	cTACE	DEB	DSM	*p*	Total	cTACE	DEB	DSM	*p*
Total	192 (100)	45 (23.4)	84 (43.7)	63 (32.8)		108 (100)	25 (23.1)	45 (41.7)	38 (35.2)		84 (100)	20 (23.8)	39 (46.4)	25 (29.8)	
Sex															
Male	151 (78.6)	36 (80.0)	63 (75.0)	52 (82.5)	0.540	84 (77.8)	19 (76.0)	33 (73.3)	32 (84.2)	0.480	67 (79.8)	17 (85.0)	30 (76.9)	20 (80.0)	0.765
Female	41 (21.4)	9 (120.0)	21 (25.0)	11 (17.5)		24 (22.2)	6 (24.0)	12 (26.7)	6 (15.8)		17 (20.2)	3 (15.0)	9 (23.1)	5 (20.0)	
Median age (range), years	65 (10–87)	62 (35–78)	63 (10–87)	66 (49–83)	0.142	60 (10–83)	60 (35–68)	58 (10–75)	62 (49–83)	0.123	70 (14–87)	66 (51–78)	70 (14–87)	72 (60–81)	0.196
Etiology of liver disease															
Alcoholic	78 (40.6)	23 (51.1)	28 (33.3)	27 (42.9)	0.622	48 (44.4)	14 (56.0)	18 (40.0)	16 (42.1)	0.396	30 (35.7)	9 (45.0)	10 (25.6)	11 (44.0)	0.725
Viral hepatitis	49 (25.5)	12 (26.7)	21 (25.0)	16 (25.4)		34 (31.5)	8 (32.0)	15 (33.3)	11 (28.9)		15 (17.9)	4 (20.0)	6 (15.4)	5 (20.0)	
Biliary disease	1 (0.5)	0 (0)	1 (1.2)	0(0)		0 (0)	0 (0)	0 (0)	0 (0)		1 (1.2)	0 (0)	1 (100)	0 (0)	
NASH	18 (9.4)	4 (8.9)	7 (8.3)	7 (11.1)		12 (11.1)	3 (12.0)	3 (6.7)	6 (15.8)		6 (7.1)	1 (5.0)	4 (10.3)	1 (4.0)	
Toxic	2 (1.0)	0 (0)	1 (1.2)	1 (1.6)		1 (0.9)	0 (0)	1 (2.2)	0 (0)		1 (1.2)	0 (0)	0 (0)	1 (100)	
Autoimmune hepatitis	2 (1.0)	1 (2.2)	1 (1.2)	0 (0)		0 (0)	0 (0)	0 (0)	0 (0)		2 (2.4)	1 (5.0)	1 (2.6)	0 (0)	
Hemochromatosis	2 (1.0)	0 (0)	1 (1.2)	0 (0)		1 (0.9)	0 (0)	0 (0)	0 (0)		1 (1.2)	0 (0)	1 (2.6)	0 (0)	
Cryptogenic	40 (20.8)	5 (11.1)	24 (28.6)	12 (19.0)		12 (11.1)	0 (0)	8 (17.8)	5 (13.2)		28 (33.3)	5 (25.0)	16 (41.0)	7 (28.0)	
ECOG															
0	91 (47.4)	18 (40.0)	43 (51.2)	30 (47.6)	0.450	67 (62.0)	14 (56.0)	31 (68.9)	22 (57.9)	0.520	24 (28.6)	4 (20.0)	12 (30.8)	8 (32.0)	0.190
1	86 (44.8)	21 (46.7)	36 (42.9)	29 (46.0)		36 (33.3)	9 (36.0)	12 (26.7)	15 (39.5)		50 (59.5)	12 (60.0)	24 (61.5)	14 (56.0)	
2	12 (6.3)	5 (11.1)	5 (6.0)	2 (3.2)		4 (3.7)	1 (4.0)	2 (4.4)	1 (2.6)		8 (9.5)	4 (20.0)	3 (7.7)	1 (4.0)	
3	3 (1.6)	1 (2.2)	0 (0)	2 (3.2)		1 (0.9)	1 (4.0)	0 (0)	0 (0)		2 (2.4)	0 (0)	0 (0)	2 (8.0)	
BCLC															
A	97 (50.5)	22 (48.9)	38 (45.2)	37 (58.7)	0.594	69 (63.9)	14 (56.0)	27 (60.0)	28 (73.7)	0.358	28 (33.3)	8 (40.0)	11 (28.2)	9 (36.0)	0.526
B	71 (37.0)	16 (35.6)	33 (39.3)	22 (34.9)		34 (31.5)	9 (36.0)	16 (35.6)	9 (23.7)		37 (44.4)	7 (35.0)	17 (43.6)	13 (52.0)	
C	15 (7.8)	4 (8.9)	9 (10.7)	2 (3.2)		1 (0.9)	0 (0)	0 (0)	1 (2.6)		14 (16.7)	4 (20.0)	9 (23.1)	1 (4.0)	
D	9 (4.7)	3 (6.7)	4 (4.8)	2 (3.2)		4 (3.7)	2 (8.0)	2 (4.4)	0 (0)		5 (6.0)	1 (5.0)	2 (5.1)	2 (8.0)	
Previous therapy															
Resection	13 (6.8)	2 (4.4)	7 (8.3)	4 (6.3)	E	7 (6.5)	0 (0)	5 (11.1)	2 (5.3)	E	6 (7.1)	2 (10.0)	2 (5.1)	2 (8.0)	E
Systemic	10 (5.2)	2 (4.4)	6 (7.1)	2 (3.2)	E	3 (2.8)	1 (4.0)	2 (4.4)	0 (0)	E	7 (8.3)	1 (14.3)	4 (10.3)	2 (8.0)	E
Local															
RFA	2 (1.0)	1 (2.2)	1 (1.2)	0 (0)	E	2 (1.8)	1 (4.0)	1 (2.2)	0	E	0 (0)	0 (0)	0 (0)	0 (0)	E
MWA	1 (0.5)	0 (0)	0 (0)	1 (1.6)		0 (0)	0 (0)	0 (0)	0 (0)		1 (1.2)	0 (0)	0 (0)	1 (4.0)	
TARE	12 (6.3)	2 (4.4)	6 (7.1)	4 (6.3)		5 (4.6)	0 (0)	2 (4.4)	3 (7.9)		7 (8.3)	2 (10.0)	4 (10.3)	1 (4.0)	
PEI	4 (2.1)	0 (0)	1 (1.2)	3 (4.8)		4 (3.7)	0 (0)	1 (2.2)	3 (7.9)		0 (0)	0 (0)	0 (0)	0 (0)	
Mean number of hepatic tumor lesions (SD)	2.2 (2.2)	2.8 (2.6)	2.3 (2.3)	1.8 (1.6)	0.109	2.3 (2.3)	2.8 (2.5)	2.6 (2.6)	1.8 (1.8)	0.185	2.1 (2.1)	3.0 (2.8)	1.9 (2.0)	1.8 (1.2)	0.112
Hepatic tumor burden															
0–25%	167 (87.0)	37 (82.2)	72 (85.7)	58 (92.1)	0.405	99 (91.7)	21 (84.0)	42 (93.3)	36 (94.7)	0.219	68 (81.0)	16 (80.0)	30 (76.9)	22 (88.0)	0.722
26–50%	23 (12.0)	8 (17.8)	11 (13.1)	4 (6.3)		8 (7.4)	4 (16.0)	3 (6.7)	1 (2.6)		15 (17.9)	4 (20.0)	8 (20.5)	3 (12.0)	
> 50%	2 (1.0)	0 (0)	1 (1.2)	1 (1.6)		1 (0.9)	0 (0)	0 (0)	1 (1.6)		1 (1.2)	0 (0)	1 (2.6)	0 (0)	
Portal vein invasion	10 (5.2)	5 (11.1)	3 (3.6)	2 (3.2)	E	5 (4.6)	3 (12.0)	1 (2.2)	1 (2.6)	E	5 (6.0)	2 (10.0)	2 (5.1)	1 (4.0)	E
Within Milan criteria	62 (32.3)	10 (22.2)	27 (32.1)	25 (39.7)	0.285	62 (57.4)	10 (40.0)	27 (60.0)	25 (65.8)	0.116	0 (0)	0 (0)	0 (0)	0 (0)	E
Mean sum of target lesion diameter (SD), mm	50.1 (28.9)	46.2 (29.4)	53.9 (29.4)	47.8 (27.7)	0.262	42.8 (23.8)	39.8 (19.6)	43.8 (22.2)	43.4 (28.0)	0.774	59.5 (32.1)	54.2 (37.3)	65.6 (32.5)	54.4 (26.4)	0.281
Mean AFP (SD), ng/ml	2299.3 (11,787.4)	1202.6 (4314.8)	4326.8 (17,828.3)	707.7 (2966.8)	0.194	769.8 (3079.2)	1060.9 (4441.9)	425.2 (975.0)	985.1 (3689.8)	0.655	4692.1 (18,314.6)	1379.7 (4288.3)	10,569.4 (27,930.0)	219.1 (431.9)	0.113
Catheter application position															
Unselective	91 (47.4)	22 (48.9)	34 (40.5)	35 (55.6)	0.101	48 (44.4)	14 (56.0)	15 (33.3)	19 (50.0)	0.356	43 (51.2)	8 (40.0)	19 (48.7)	16 (64.0)	0.123
Selective	57 (29.7)	17 (37.8)	24 (28.6)	16 (25.4)		32 (29.6)	6 (24.0)	15 (33.3)	11 (34.4)		25 (29.8)	11 (55.0)	9 (28.2)	5 (20.0)	
Superselective	44 (22.9)	6 (13.3)	26 (31.0)	12 (19.0)		28 (25.9)	5 (20.0)	15 (33.3)	8 (21.1)		16 (19.0)	1 (5.0)	11 (28.2)	4 (16.0)	

Values denote *n* (%), mean (SD) or median (range)

ANOVA for continuous variables, Chi-Square test for categorial variables (E, not calculated due to low sample size)

Abbreviations: *AFP* alpha-fetoprotein, *BCLC* Barcelona clinic liver cancer (stage), *cTACE* conventional transarterial chemoembolization, *DEB* drug-eluting bead, *DSM* degradable starch microsphere, *ECOG* Eastern Cooperative Oncology Group (performance status), *MWA* microwave ablation, *NASH* non-alcoholic steatohepatitis, *PEI* percutaneous ethanol injection, *RFA* radiofrequency ablation, *TACE* transarterial chemoembolization, *TARE* transarterial radioembolization

### Changes in liver function and side effects

Changes in liver function parameters are shown in Table 2. All three TACE techniques were associated with transient changes in liver function. 2 days post-TACE, especially GOT and GPT were elevated with no significant differences between the three TACE techniques. Slight increases in bilirubin [DEB, 0.6 (1.2) mg/dl vs. cTACE, −0.5 (1.4) mg/dl and DSM, 0.2 (0.9) mg/dl; *p* = 0.025] in the palliative subgroup and lactate dehydrogenase [DEB, 18.7 (89.8) U/l vs. DSM, −19.7 (65.9) U/l; *p* = 0.044] in the entire cohort were more pronounced with the DEB technique at 1-month follow-up.

**Table 2:** Changes in liver function and side effects of 1st TACE

	Entire cohort				Bridging/downstaging				Palliative			
	cTACE	DEB	DSM	*p*	cTACE	DEB	DSM	*p*	cTACE	DEB	DSM	*p*
Δ Liver function (2 days post TACE)												
INR	0 (0.1)	0 (0.1)	0 (0.2)	0.743	0 (0.8)	0 (0.6)	0 (0.6)	0.525	0 (0.5)	0 (0.2)	0 (0.4)	0.498
Bilirubin [mg/dl]	0 (0.7)	0 (0.4)	0 (0.5)	0.935	−0.1 (0.7)	0 (0.4)	0 (0.5)	0.591	0.2 (0.6)	0 (0.3)	0.1 (0.5)	0.514
GOT [U/l]	24.8 (88.5)	54.9 (116.7)	52.0 (115.2)	0.378	28.8 (110.2)	23.6 (60.9)	42.5 (117.6)	0.683	18.4 (37.0)	91.3 (151.9)	66.0 (112.6)	0.186
GPT [U/l]	19.6 (58.6)	18.4 (46.7)	22.3 (56.8)	0.912	26.5 (72.5)	10.8 (29.1)	17.7 (61.2)	0.518	7.7 (15.5)	27.3 (60.5)	29.1 (50.3)	0.417
GGT [U/l]	−7.8 (21.4)	−2.3 (50.5)	−12.1 (35.5)	0.404	−8.7 (22.2)	1.3 (47.6)	−15.6 (40.8)	0.21	−6.2 (20.6)	−6.5 (54.0)	−7.1 (26.4)	0.998
ALP [U/l]	−19.0 (39.1)	−5.1 (58.1)	−8.5 (18.4)	0.404	−27.6 (44.9)	−10.8 (29.0)	−9.6 (18.2)	0.121	−0.8 (8.7)	1.6 (80.3)	−7.1 (19.2)	0.893
LDH [U/l]	−3.9 (103.1)	13.6 (147.1)	30.0 (65.7)	0.670	−6.1 (100.1)	4.5 (62.0)	13.4 (62.9)	0.816	3.0 (128.4)	24.8 (213.2)	52.2 (66.2)	0.870
Δ Liver function (1-month follow-up)												
INR	0 (0.1)	1.3 (11.2)	2.1 (15.7)	0.683	0 (0.2)	2.4 (15.2)	3.4 (20.2)	0.724	0 (0.1)	0 (0.1)	0.1 (0.3)	0.355
Bilirubin [mg/dl]	−0.1 (1.0)	0.2 (1.0)	0.1 (0.8)	0.279	0.2 (0.6)	−0.1 (0.6)	0 (0.7)	0.271	**−0.5 (1.4)**^**a**^	**0.6 (1.2)**^**b**^	**0.2 (0.9)**^**c**^	**0.025**
GOT [U/l]	−2.0 (47.9)	15.8 (130.8)	−3.9 (26.2)	0.383	−2.1 (56.1)	−5.6 (23.6)	−4.8 (26.9)	0.927	−1.9 (32.8)	40.2 (188.3)	−2.7 (25.7)	0.378
GPT [U/l]	5.0 (21.4)	−0.6 (32.1)	−3.8 (16.4)	0.240	7.0 (24.7)	−2.1 (26.7)	−4.5 (17.7)	0.155	1.8 (16.4)	1.0 (37.4)	−2.8 (14.3)	0.845
GGT [U/l]	4.0 (60.5)	20.5 (152.1)	−20.0 (110.0)	0.153	4.4 (40.7)	14.6 (65.5)	−32.8 (121.8)	0.050	3.4 (84.4)	26.9 (210.1)	−1.1 (88.6)	0.763
ALP [U/l]	8.5 (36.7)	27.9 (98.5)	6.7 (54.1)	0.216	14.6 (39.8)	6.1 (53.4)	11.5 (64.9)	0.830	−1.9 (29.0)	54.1 (130.4)	−1.2 (28.9)	0.054
LDH [U/l]	**15.2 (62.9)**^**a**^	**18.7 (89.8)**^**a**^	**−19.7 (65.0)**^**b**^	**0.044**	15.7 (65.5)	−3.0 (41.2)	−12.5 (71.1)	0.272	14.3 (60.4)	43.0 (120.0)	−31.2 (54.4)	0.063
Mean inpatient stay (SD), d	4.8 (3.2)	4.4 (3.3)	4.1 (2.8)	0.507	4.8 (2.5)	4.5 (3.3)	4.5 (3.3)	0.907	4.7 (4.0)	4.2 (3.3)	3.3 (1.6)	0.324
Prolonged hospital stay (> 48 h beyond normal course)	12 (26.7)	19 (22.6)	14 (22.2)	0.842	9 (36.0)	9 (20.0)	11 (28.9)	0.329	3 (15.0)	10 (25.6)	3 (12.0)	0.347
Post-embolization syndrome	2 (4.3)	9 (10.7)	2 (3.2)	E	0 (0)	2 (4.7)	0 (0)	E	2 (10.0)	7 (18.0)	2 (8.0)	E
CIRSE AE grade 1–3	12 (26.7)	25 (29.7)	14 (22.2)	0.318	9 (36.0)	10 (22.2)	11 (28.9)	0.214	**3 (15.0)**^**a**^	**15 (38.5)**^**b**^	**3 (12.0)**^**a**^	**0.038**

Values denote n (%) or mean (SD)

ANOVA for continuous variables (Sidak post-hoc test: Values with the same letter superscripted do not vary significantly), Chi-Square test for categorial variables (Bonferroni post-hoc test: Values with the same letter superscripted do not vary significantly; E, not calculated due to low sample size)

Abbreviations: *AE* adverse events, *ALP* alkaline phosphatase, *cTACE* conventional transarterial chemoembolization, *DEB* drug-eluting bead, *DSM* degradable starch microsphere, *GGT* gamma-glutamyl transferase, *GOT* aspartate aminotransferase, *GPT* alanine aminotransferase, *INR* international normalized ratio, *LDH* lactate dehydrogenase, *TACE* transarterial chemoembolization

The incidence of CIRSE AE Grade 1–3 was significantly higher in DEB-TACE than in cTACE or DSM-TACE in the palliative subgroup (*p* = 0.038). Importantly, we did not observe any CIRSE grade 4–6 AE/SAE in our study following cTACE, DEB-TACE, or DSM-TACE.

### Tumor response after first TACE

Table 3 summarizes the tumor response at 4–6 weeks after the first TACE using mRECIST criteria. In the entire cohort, DEB-TACE and DSM-TACE showed significantly higher disease control rates (complete and partial response, stable disease) compared to cTACE [TACE lesions: DEB, *n *= 81 (96.4%) and DSM, *n *= 60 (95.2%) vs. cTACE, *n *= 37 (82.2%); *p* = 0.008]. Target lesions: DEB, *n *= 82 (97.6%) and DSM, *n *= 60 (95.2%) vs. cTACE, *n *= 38 (84.4%); *p* = 0.011]. This trend was also evident in the B/D subgroup: Here, the DEB technique was superior to the DSM technique compared to cTACE in terms of TACE lesion response [DEB, *n *= 27 (60.0%) vs. cTACE, *n *= 7 (28.0%); *p* = 0.034], target lesion DCR [DEB, *n *= 44 (97.8%) vs. cTACE, *n *= 19 (76.0%); *p* = 0.005], and overall DCR [DEB, *n *= 39 (86.7%) vs. cTACE, *n *= 15 (60.0%); *p* = 0.029]. In the palliative subgroup, there were no differences between the different techniques.

**Table 3:** Response at 1-month follow-up

	Entire cohort				Bridging/downstaging				Palliative			
	cTACE	DEB	DSM	*p*	cTACE	DEB	DSM	*p*	cTACE	DEB	DSM	*p*
TACE lesions												
CR	4 (8.9)	15 (17.9)	14 (22.2)	0.190	2 (8.0)	11 (24.4)	12 (31.6)	0.091	2 (10.0)	4 (10.3)	2 (8.0)	0.953
Response (CR/PR)	15 (33.3)	45 (53.6)	28 (44.4)	0.086	**7 (28.0)**^**a**^	**27 (60.0)**^**b**^	**20 (52.6)**^**a,b**^	**0.034**	8 (40.0)	18 (46.2)	8 (32.0)	0.530
DCR (CR/PR/SD)	**37 (82.2)**^**a**^	**81 (96.4)**^**b**^	**60 (95.2)**^**a,b**^	**0.008**	**18 (72.0)**^**a**^	**43 (95.6)**^**b**^	**36 (94.7)**^**b**^	**0.004**	19 (95.0)	38 (97.4)	24 (96.0)	0.884
PD	**8 (17.8)**^**a**^	**3 (3.6)**^**b**^	**3 (4.8)**^**a,b**^		**7 (28.0)**^**a**^	**2 (4.4)**^**b**^	**2 (5.3)**^**b**^		1 (5.0)	1 (2.6)	1 (4.0)	
Target (mRECIST) lesions												
CR	3 (6.7)	15 (17.9)	12 (19.0)	0.164	2 (8.0)	11 (24.4)	11 (28.9)	0.132	1 (5.0)	4 (10.3)	1 (4.0)	0.583
Response (CR/PR)	13 (28.9)	39 (46.4)	28 (44.4)	0.135	7 (28.0)	24 (53.3)	20 (52.6)	0.09	6 (30.0)	15 (38.5)	8 (32.0)	0.771
DCR (CR/PR/SD)	**38 (84.4)**^**a**^	**82 (97.6)**^**b**^	**60 (95.2)**^**a,b**^	**0.011**	**19 (76.0)**^**a**^	**44 (97.8)**^**b**^	**36 (94.7)**^**a,b**^	**0.005**	19 (95.0)	38 (97.4)	24 (96.0)	0.884
PD	**7 (15.6)**^**a**^	**2 (2.4)**^**b**^	**3 (4.8)**^**a,b**^		**6 (24.0)**^**a**^	**1 (2.2)**^**b**^	**2 (5.3)**^**a,b**^		1 (5.0)	1 (2.6)	1 (4.0)	
Overall (mRECIST)												
CR	2 (4.4)	12 (14.3)	10 (5.2)	0.168	2 (8.0)	9 (20.0)	9 (23.7)	0.277	0 (0)	3 (7.7)	1 (4.0)	0.413
Response (CR/PR)	12 (26.7)	35 (41.7)	26 (41.3)	0.200	7 (28.0)	22 (48.9)	18 (47.4)	0.201	5 (25.0)	13 (33.3)	8 (32.0)	0.799
DCR (CR/PR/SD)	32 (71.1)	72 (85.7)	52 (82.5)	0.122	**15 (60.0)**^**a**^	**39 (86.7)**^**b**^	**31 (81.6)**^**a,b**^	**0.029**	17 (85.0)	33 (84.6)	21 (84.0)	0.996
PD	13 (28.9)	12 (14.3)	11 (17.5)		**10 (40.0)**^**a**^	**6 (13.3)**^**b**^	**7 (18.4)**^**a,b**^		3 (15.0)	6 (15.4)	4 (16.0)	

Values denote *n* (%)

Chi-Square test for categorial variables (Bonferroni post-hoc test: Values with the same letter superscripted do not vary significantly)

Abbreviations: *CR* complete response, *cTACE* conventional transarterial chemoembolization, *DCR* disease control rate, *DEB* drug-eluting bead, *DSM* degradable starch microsphere, *mRECIST* modified response evaluation criteria in solid tumors, *PR* partial response, *SD* stable disease, *TACE* transarterial chemoembolization

### Progression-free survival

**Fig. 2:**
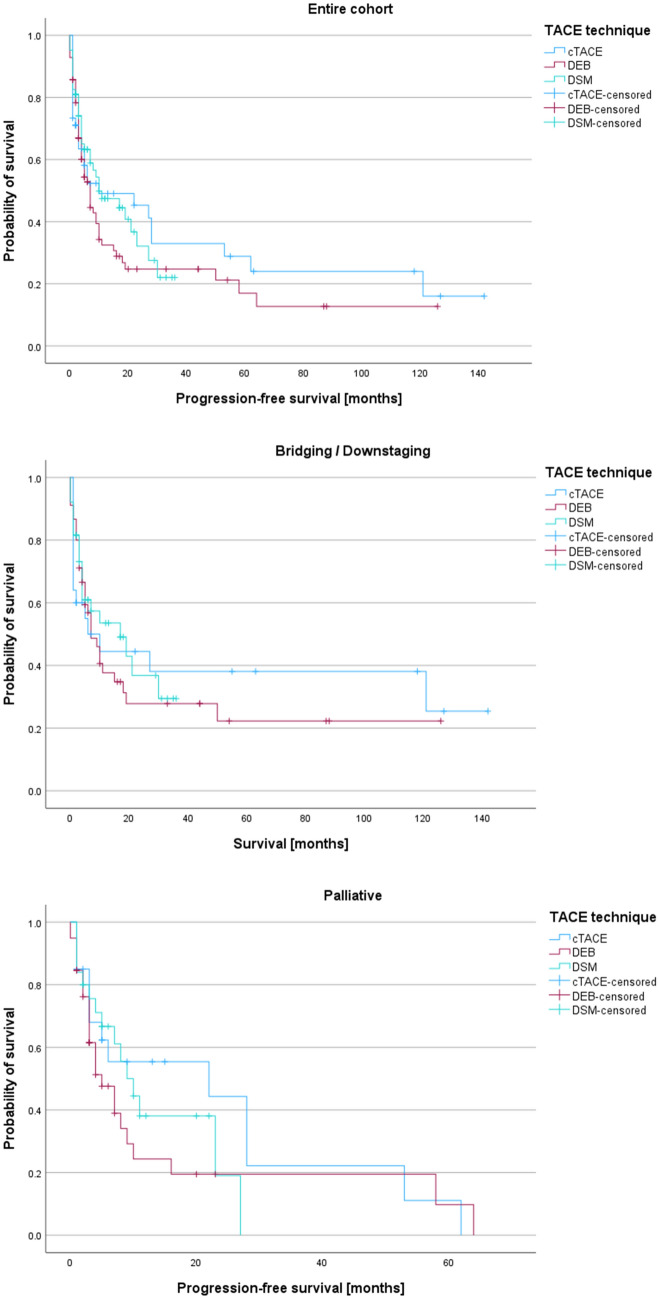
Survival analysis of the entire cohort and B/D- and palliative subgroup. There was no significant difference in PFS between the groups (see also Table 4). Abbreviations: cTACE, conventional transarterial chemoembolization; DEB, drug-eluting beads; DSM, degradable starch microsphere; TACE, transarterial chemoembolization

Table 4 and Fig. 2 display the Kaplan–Meier data for PFS in the three TACE techniques. There was no significant difference in PFS between the groups [median PFS (months), first TACE technique: cTACE, 10.0 vs. DEB, 7.0 vs. DSM, 10.0; *p* = 0.436].

**Table 4:** Progression-free survival after 1st TACE

	Median PFS, months*	95% CI, months*	p**
Entire cohort			
cTACE	10.0	0–30.4	0.436
DEB	7.0	4.7–9.2	
DSM	10.0	0–20.1	
Bridging/downstaging			
cTACE	6.0	0–15.6	0.785
DEB	7.0	3.2–10.7	
DSM	17.0	2.2–31.8	
Palliative			
cTACE	22.0	0–61.0	0.478
DEB	5.0	1.9–8.0	
DSM	9.0	5.1–12.9	

Values denote median (95% CI)

*Kaplan–Meier estimator. **Log-rank-test

Abbreviations: *CI* confidence interval, *cTACE* conventional transarterial chemoembolization, *DEB* drug-eluting bead, *DSM* degradable starch microsphere, *PFS* progression-free survival, *TACE* transarterial chemoembolization

### Listing and liver transplantation success rate

Table 5 shows the rates of successful listing after first TACE and in the case of listing for successful liver transplantation. Here, fewer patients were listed after first DSM-TACE, although more of those listed after first DSM-TACE were transplanted, with a relatively short median waiting time although without significant differences between the groups.

**Table 5:** Success rate of Bridging/downstaging

	Bridging/downstaging subgroup			
	cTACE	DEB	DSM	*p*
LT listing after 1st TACE	22 (91.7)	40 (88.9)	28 (73.7)	0.087
LT after 1st TACE	11 (50.0)	27 (67.5)	22 (78.6)	0.183
Median time to LT after 1st TACE (range), m	12 (1–14)	8 (1–22)	4.5 (1–32)	0.316

Values denote *n* (%) or median (range)

ANOVA for continuous variables, Chi-Square test for categorial variables

Abbreviations: *cTACE* conventional transarterial chemoembolization, *DEB* drug-eluting bead, *DSM* degradable starch microsphere, *LT* liver transplantation, *TACE* transarterial chemoembolization

## Discussion

TACE has become a cornerstone in the management of HCC (Lucatelli et al. 2021; Reig et al. 2022; Claasen et al. 2023). However, current guidelines do not uniformly recommend which TACE technique is favorable for specific patient and tumor characteristics, leaving the decision of which technique to use to the interventional radiologist on a case-by-case basis (Marrero et al. 2018; Angeli et al. 2018; Lucatelli et al. 2021). This fact and the lack of standardization in TACE make comparative studies to other anticancer treatments cumbersome, and clear evidence for superiority or non-superiority of certain TACE techniques is therefore needed. This study directly compared three TACE techniques—cTACE, DEB-TACE, and DSM-TACE—in a single-center cohort of HCC patients with subgroup analysis regarding the indication for TACE, aiming to shed light on their relative merits and drawbacks.

First, our study investigates the impact of TACE techniques on liver function. While all TACE techniques resulted in transient, but not significant changes in liver function 48 to 72 h post-TACE, DEB-TACE induced slight, but significant increases in bilirubin in the palliative subgroup and in lactate dehydrogenase in the entire cohort at 4 to 6 weeks post-TACE, suggesting a slight, but prolonged impairment of liver function. However, in a meta-analysis by Wang et al. (2023) evaluating the efficacy and safety of DEB vs. cTACE in patients with unresectable HCC, DEB-TACE was not superior to cTACE with respect to adverse events (liver absence, liver failure, post-embolization syndrome, fatigue). In a comparable study design, Mohr et al. (2022) assessed clinical effects and safety endpoints of the same three TACE techniques in a single-center retrospective analysis of 148 patients. While their bridging data set similarly showed no differences in liver function with respect to the TACE technique used, in contrast to our results, their palliative subgroup showed a significant increase in GOT for cTACE. In our study, we report the adverse events (AE) data according to the Cardiovascular and Interventional Radiological Society of Europe (CIRSE) Quality Assurance Document and Standards for Classification of Complications (The CIRSE Classification System) (Filippiadis et al. 2017). Although post-embolization syndrome is an expected toxicity of TACE, it should not be regarded as a complication according to CIRSE expert consensus (De Baere et al. 2016). However, in our study, we report the incidence of post-embolization syndrome beyond the normal post-procedure course requiring for additional therapy as we are convinced that this side effect of TACE procedures should be considered when treating patients, especially in the potentially repetitive setting of TACE cycles (CIRSE grade 2–3). We would like to emphasize that data analysis showed that the incidence of CIRSE AE Grade 1–3 was significantly higher in DEB-TACE than in cTACE or DSM-TACE in the palliative subgroup (*p* = 0.038). In contrast to our work and the CIRSE recommendations, Mohr et al. (2022) evaluated expected clinical symptoms after TACE, such as fever, nausea, and abdominal pain, and observed a higher incidence of nausea after cTACE in their palliative dataset. Other comparative studies on the safety of DSM-TACE are rare, which underscores the value of our study. In a multicenter study analyzing the efficacy and safety of DSM-TACE for patients with HCC with a high tumor burden ineligible for or failing other palliative therapies, DSM-TACE was well tolerated with no major clinical adverse events and preserved liver function, comparable to our results (Ludwig et al. 2021).

Second, our study highlights that DEB-TACE and DSM-TACE are associated with better disease control rates compared to cTACE, as evidenced by higher rates of complete and partial responses and stable disease according to mRECIST criteria at 4–6 weeks after TACE. These results were particularly observed in the B/D subgroup, here even with a slight, but significant advantage for DEB over DSM compared to cTACE, while there were no differences in the subgroup analysis of Mohr et al. (2022). Further studies comparing treatment response in a single-center cohort with respect to all three techniques are lacking. Two randomized controlled trials comparing cTACE with DEB-TACE: PRECISION V, a multicenter, prospective, randomized phase II study including 212 patients and PRECISION Italia, a multicenter, prospective, randomized active-controlled study including 177 patients (Lammer et al. 2010; Golfieri et al. 2014). The primary efficacy endpoint of PRECISION V was the 6-month follow-up, while the DEB arm showed higher response and disease control rates compared to the cTACE arm (52% vs. 44%, and 63% vs. 52%, respectively), although not statistically superior (*p* = 0.11). In PRECISION Italia, no differences in local and overall tumor responses were observed between the arms at 1, 3, and 6 months. A meta-analysis by Facciorusso et al. (2016) confirmed these findings with only a non-significant trend in favor of DEB-TACE. Limited evidence exists for the efficacy of DSM-TACE compared to other TACE techniques, especially considering the indication for TACE. In a prospective randomized study of 61 patients with BCLC stage B HCC who underwent TACE as a bridging treatment, Vogl et al. (2021) demonstrated a significant benefit in tumor response with DSM-TACE compared to cTACE at 1-month follow-up. These results are consistent with our subgroup analysis showing superiority of DSM-TACE over cTACE. These results, with an albeit slight superiority of DEB and DSM over cTACE, are accompanied by a prospective pilot study by Schicho et al. (2016) examining the impact of different TACE techniques on a vascular endothelial growth factor (VEGF)-dependent neo-angiogenic response. This response is caused by TACE-induced ischemia and reperfusion, which limits its effectiveness because VEGF is known to promote tumor growth via neo-angiogenesis, metastatic seeding and cancer cell migration. In their study, cTACE induced a marked VEGF response in contrast to DEB-TACE and DSM-TACE. Schicho et al. (2016) hypothesized that lipiodol alone, without defined particle size, allows a sustained but dynamic situation of tissue hypoxia followed by continuous reperfusion, in contrast to permanent embolic DEB or transient embolic DSM. These results might be an explanation for our findings and could support the use of DEB or DSM-TACE as the first causal treatment for VEGF overexpression.

Third, there was no significant difference in PFS between the different TACE groups. These results are consistent with the PRECISION Italia study and a median PFS of 9 months in both the DEB and cTACE arms (*p* = 0.766), while Mohr et al. and PRECISION V did not perform a survival analysis (Lammer et al. 2010; Golfieri et al. 2014; Mohr et al. 2022). However, non-significant trends in favor of DEB-TACE compared to cTACE were observed as for 1-year (*p* = 0.25), 2-year (*p* = 0.13), and 3-year survival (*p* = 0.06) in the meta-analysis by Facciorusso et al. (2016). In the study by Vogl et al. (2021) comparing DSM- and cTACE as bridging treatment, no survival benefit was observed.

This study is limited by its retrospective nature and the heterogeneity of the patient cohort analyzed, which limits its generalizability. Although we excluded a relatively large number of patients to obtain an unbiased follow-up at 4–6 weeks, the subsequent follow-up data were relatively heterogeneous, especially with respect to different TACE and intermediate therapies. This can be seen from the discrepancy between PFS and ORR and most likely explained by a bias due to different follow-up periods for the three TACE techniques in the retrospective setting (since first cTACE, then DEB-TACE and last DSM-TACE was implemented at the study center), although the PFS data did not reach the significance level in the group comparison. Therefore, we believe that ORR at 4–6 weeks is the more objective outcome parameter and it was important for us to exclude patients who had already received a second TACE, e.g., another technique, or another local ablative or systemic therapy between TACE and the first follow-up after 4–6 weeks. In our real-life setting, where patients typically receive multiple sessions of different techniques and other non-TACE interim therapies, we wanted to provide a structure that is otherwise only possible in a prospective setting. We are convinced that the chosen retrospective design is best suited to address the issue of which TACE technique should first be preferably used in which cohort of HCC patients. Future studies should address a lesion- or procedure-based, integrated diagnostic approach in which TACE treatment is tailored to individual patient, tumor, and imaging characteristics.

In conclusion, our study provides important perspectives in the decision-making for a specific TACE technique: Both DEB-TACE and DSM-TACE showed better response and disease control rates. However, DEB-TACE was associated with prolonged effects on liver function and side effects, so patients with impaired liver function should be more strictly selected, especially in the palliative subgroup.

## Data Availability

No datasets were generated or analysed during the current study.
